# Meeting of Dentists in Syracuse, N. Y.

**Published:** 1863-06

**Authors:** S. B. Palmer


### Proceedings of Societies.

---

At a meeting of the Dentists from Onondaga and adjoining counties, held in Syracuse, March 2nd, 1863, for the purpose of establishing a uniform fee list for dental operation, a resolution was passed to form a Dental Association in Central New York, to embrace within its limits that portion of the State bounded on the north and south by the State lines. On the west by Cayuga Lake and the eastern boundry of the "Dental Association of Western New York." On the east, as far as practical, the boundary not there determined.

Notices were accordingly sent to all Dentists, whose names could be obtained in the vicinity, calling a meeting, to be held in Syracuse, March 23. The call was responded to and a goodly number of dentists were present. The meeting proceeded to form a permanent organization known by the name of The Central New York Dental Association.

The objects of the Association are to "cultivate the science and art of dentistry, and all its collateral branches, to elevate and sustain the professional character of dentists, to promote among them mutual improvement, social intercourse and good feeling, and to collectively represent and have cognizance of the common interests of the dental profession" in Central New York.

The following were elected officers to serve one year or until their successors were elected.

Dr. D. S. Ball, of Auburn, President; Dr. D. W. Perkins, of Baldwinrith, Vice President; Dr. S. B. Palmer, of Tully, Secretary; Dr. A. T. Smith, of Syracuse, Corresponding Secretary; Dr. E. M. Skinner, of Syracuse, Treasurer.

A Committee of dentists was appointed to visit distant localities and urge attendance at the next meeting which was appointed in the same place, April 20th.

The second meeting of the Association was well attended. A Constitution and By-Laws were presented, considered and adopted.

The Association is to hold two regular meetings in each year at such place as the last meeting shall designate, the annual meeting to be held on the second Tuesday in May; the semi-annual on the second Tuesday in November.

All practicing dentists within the specified limits of the Association in good standing "shall be eligible to membership and may become members by signing the Constitution and paying an initiation fee."

The third meeting of the Association was held in Auburn, May 12th, much interest was manifested. New members were received. An address was read by S. B. Palmer of Tully, setting forth the advantages of associated effort, the claims of the profession upon its patrons, also claims of the patient upon the profession. An essayist was appointed to prepare a paper to open discussion at next meeting; subject, "The importance of preserving natural teeth and the best means to be used to accomplish that object.

Instructions were given to the Secretary to prepare a report of the organization and its designs at the several meetings, and forward the same, together with the address to the Dental Register of the West and the Dental Cosmos. The happy and beneficial influence of social intercourse was plainly manifested in the removing of misplaced professional jealousies, the renewal of old acquaintances and the formation of many agreeable new ones.

The Association adjourned to meet in Syracuse on the second Tuesday of November, 1863.

S. B. PALMER,
*Secretary.*

